# Recent advances in modulators of circadian rhythms: an update and perspective

**DOI:** 10.1080/14756366.2020.1772249

**Published:** 2020-06-08

**Authors:** Shenzhen Huang, Xinwei Jiao, Dingli Lu, Xiaoting Pei, Di Qi, Zhijie Li

**Affiliations:** Henan Eye Institute, Henan Eye Hospital and Henan Key Laboratory of Ophthalmology and Visual Science, Henan Provincial People’s Hospital, People’s Hospital of Zhengzhou University, People’s Hospital of Henan University, Zhengzhou, China

**Keywords:** Circadian rhythm, clock proteins, post-modification targets, small-molecule modulators, circadian rhythm-related disease

## Abstract

Circadian rhythm is a universal life phenomenon that plays an important role in maintaining the multiple physiological functions and regulating the adaptability to internal and external environments of flora and fauna. Circadian alignment in humans has the greatest effect on human health, and circadian misalignment is closely associated with increased risk for metabolic syndrome, cardiovascular diseases, neurological diseases, immune diseases, cancer, sleep disorders, and ophthalmic diseases. The recent description of clock proteins and related post-modification targets was involved in several diseases, and numerous lines of evidence are emerging that small molecule modulators of circadian rhythms can be used to rectify circadian disorder. Herein, we attempt to update the disclosures about the modulators targeting core clock proteins and related post-modification targets, as well as the relationship between circadian rhythm disorders and human health as well as the therapeutic role and prospect of these small molecule modulators in circadian rhythm related disease.

## 1. Introduction

**Figure 1.**
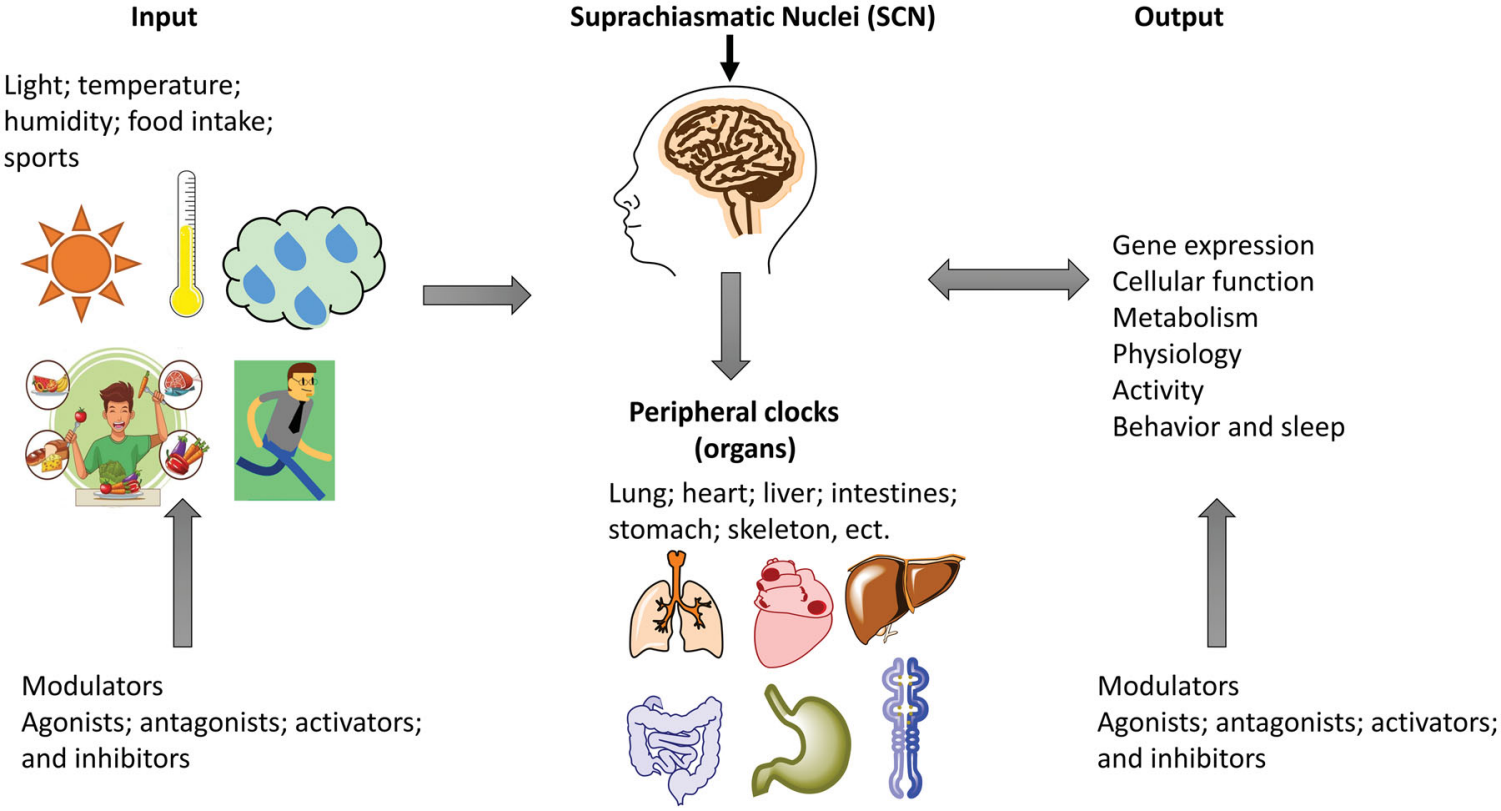
The physiological basis for the generation and maintenance of mammalian circadian rhythm. Reproduced from Chen et al.^19^

**Figure 2.**
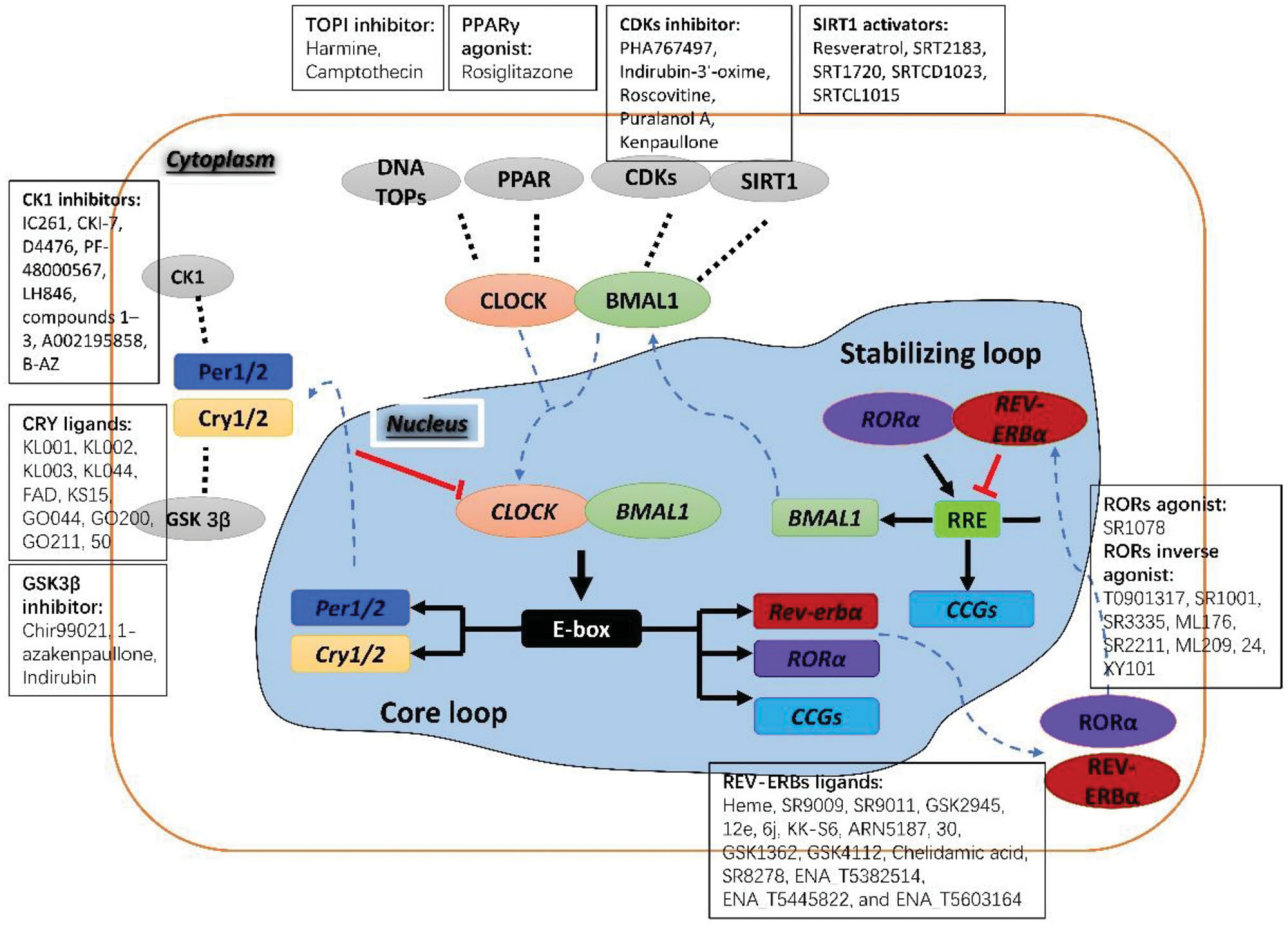
Molecular clock loops and their potential targets with representative small molecule modulators. CLOCK: circadian locomotor output cycles kaput; BMAL1: brain and muscle ARNT-like 1; CRY: cryptochrome; PER: period; ROR: RAR-related orphan receptor; RRE: retinoic acid receptor-related orphan receptor binding element; CCGs: clock-controlled genes; CK1: casein kinase 1; CDKs: cyclin-dependent kinases; GSK3β: glycogen synthase kinase 3β; SIRT1: silent information regulator 1; PPARγ: peroxisome proliferator-activated receptor γ; DNA TOPs: DNA topoisomerases. Reproduced from He and Chen^49^. Copyright 2016 American Chemical Society.

Circadian rhythm is the result of natural selection during the long-term evolution of organisms, enabling organisms to better adapt to changes in the external environment^1^^,^^2^. Various behaviours and physiological functions of the body show obvious circadian rhythms, such as the sleep-wake cycle^3^^,^^4^, food intake and other autonomous activities^5^, as well as physiological activities including blood pressure^6^, blood lipids, coagulation-fibrinolysis balance, heart rate^7^^,^^8^, body temperature^9^, locomotor activity^10^^,^^11^, hormone levels^12^, cell metabolism^13^, and cell proliferation^14^^,^^15^. The generation, maintenance, and regulation of circadian rhythms depend on the synergy of the circadian clock system, circadian input system, and circadian output system at the overall level (Figure 1) and at the cellular level, relying on the precise regulation of the endogenous circadian clock gene network (Figure 2). Any abnormalities in these intrinsic rhythms can cause disturbances in the circadian rhythm.

The physiological basis for the generation and maintenance of circadian rhythms comprises the central and peripheral circadian clock systems, rhythm input systems, and rhythm output systems. The rhythm input system senses and transmits environmental synchronisation signals represented by light signals to the central circadian clock system. The central biological clock system acts as the circadian rhythm pacemaker through the output system to transmit the generated rhythm signals to the periphery, and cooperates with the endogenous biological clock system of the peripheral organs to maintain the physiological activity of the body^16^ (Figure 1). The circadian clock system is composed of the central circadian clock and the peripheral circadian clock. In mammals, the apex of this system is the suprachiasmatic nuclei (SCN) master pacemaker, which is considered the central or master clock^17^. The SCN integrates the environmental time information (primarily light) via the retina to revamp or entrain its phase, and then mastermind other oscillators in extra-SCN brain regions and peripheral organs^18^^,^^19^. The rhythm output system is regulated by SCN, which can regulate gene expression, cellular function, metabolism, physiology, activity, behaviour, and sleep-wake cycles^20^. Additionally, the rhythm output systems in turn can affect the SCN master pacemaker^19^^,^^20^. For example, the arrhythmic food intake, excessive exercises, and sleep/circadian disorders affect SCN by remodel clock-controlled circuit^21–24^.

Circadian rhythm production and maintenance are regulated by circadian clock genes. The molecular mechanism of the mammalian circadian clock is produced by a cell-autonomous feedback loop^25–27^. The periodic oscillation of circadian rhythm depends on the precise regulation of the circadian clock gene and the clock-controlled gene regulatory network, including transcriptional-translational feedback loops^28^^,^^29^ and the non-transcription mechanism of post-translational modification^30^^,^^31^. As shown in Figure 2, the transcriptional-translational feedback loops include a core loop and a secondary stabilisation loop.

In mammals, the transcription factors circadian locomotor output cycles kaput (CLOCK) and brain and muscle ARNT-like 1 (BMAL1) form a heterodimer, which binds to E-box enhancers to activate the target gene transcription of circadian clock gene *Period* (including *Per1* and *Per2*) and *Cryptochrome* (including *Cry*1 and *Cry2*). When PER and CRY proteins accumulate to a certain extent, they could be further transferred from the cytoplasm to the nucleus, and the PER/CRY heterodimer as a negative regulator directly interacts with CLOCK/BMAL1 to inhibit its transcriptional activity^32^. In the stabilisation loop, the CLOCK/BMAL1 heterodimer can also induce the expression of nuclear receptors *REV-ERBα* and *RORα*. As a negative regulator, REV-ERBα can bind to the retinoic acid receptor-related orphan receptor binding element (RRE) (sequence AGGTCA) in the *BMAL1* promoter region and block the transcription of *BMAL1*^33^. Conversely, *RORα* can be used as a positive regulator to bind to the RRE of the *BMAL1* promoter region to promote the transcription of *BMAL1*, thereby forming an auxiliary loop for the transcription and translation oscillations of the circadian clock gene^34^. However, beyond that, post-translational modifications (phosphorylation/dephosphorylation, acetylation/deacetylation, etc.) and degradation (ubiquitination/proteasome pathway) of various circadian proteins enable fine-tuning of the transcriptional-translational feedback loops (such as adjusting the expression phase and the period of oscillation), so it can also play an important role in the cyclical cycle of circadian rhythms^30^^,^^31^. For example, PER and CRY proteins can be phosphorylated by casein kinase 1ε (CK1ε)/casein kinase 1ε (CK1δ), which affects the increase in the continuous length of the cycle^35^. Silent information regulator 1 (SIRT1) regulates the expression of the clock gene *BMAL1*, *Cry1*, and *Per2* by interacting with the CLOCK/BMALI complex and catalysing the deacetylation and degradation of the PER protein^36^.

Circadian alignment in humans has great effect on human health, and circadian misalignment has been involved in metabolic syndrome^37^^,^^38^, cardiovascular diseases^39^^,^^40^, acute lung injury and inflammation^41^, cancer^42–44^, neurological diseases^45^^,^^46^, and immune diseases^47^^,^^48^. While accumulating evidence indicates that small molecule modulators of circadian rhythms can be used to rectify circadian disorder^18^^,^^49–51^, in this review, we pay attention to the recent progress of small molecule modulators targeting core clock proteins (such as CRYs, REV-ERBs, and RORs) and related post-modification targets (such as casein kinase 1 (CK1), cyclin-dependent kinases (CDKs), glycogen synthase kinase 3 (GSK3), cdc2-like kinase 1 (CLK1), breakpoint cluster region-Abelson tyrosine kinase (BCR-ABL), and silent information regulator 1 (SIRT1)), as well as the relationship between circadian rhythm disorders and human health and the therapeutic role and prospect of these small molecules in circadian rhythm related disease.

## 2. Overview of modulators targeting circadian rhythms

As mentioned above, circadian rhythms are associated with a variety of biological functions and biological dysfunctions. Efforts to develop initial modulators have focussed on the circadian clock, and modulators including endogenous and synthetic compounds have been discovered. The identified modulators can be classified into two broad categories, which are targeting core clock proteins and other or unknown targets.

### 2.1. Small molecule modulators of core clock proteins

#### 2.1.1. Modulators for CRYs

**Figure 3.**
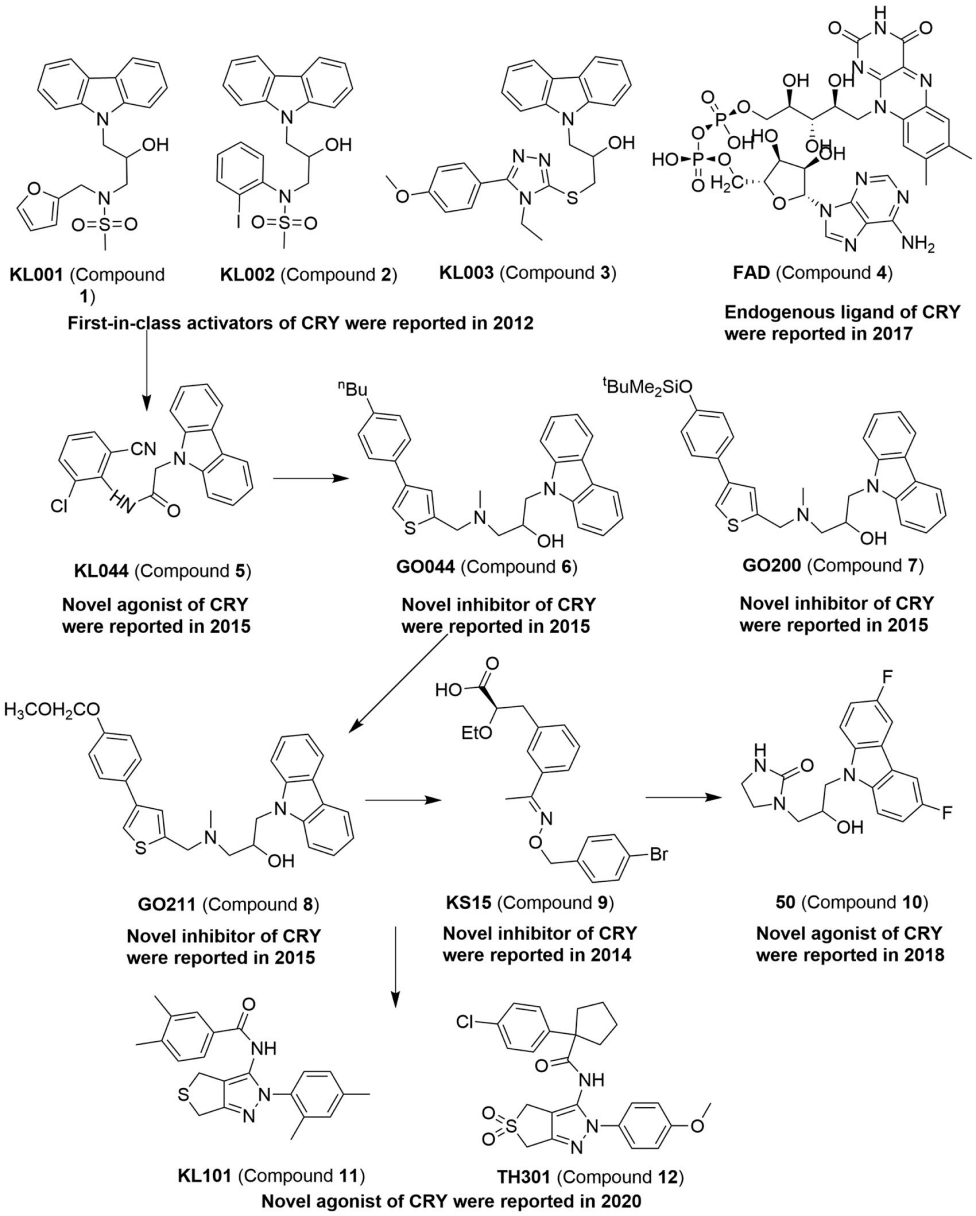
The structure of modulators targeting CRYs.

Compound **1** (Table 1 and Figure 3), the first-in-class small molecules, comprise carbazole derivative and an activator of cryptochromes (CRYs) ^52^. The carbazole derivatives, such as compound **1**–**3**, can specifically interact with CRY1 and CRY2 and cause period lengthening and amplitude reduction in a dose-dependent manner in stable U2OS reporter cell lines harbouring *Bmal1-dLuc* or *Per2-dLuc*. Compound **1** can inhibit glucagon-induced gluconeogenesis by stabilising the CRYs. The co-crystal structure of murine CRY2 PHR core domain (1–512) with compound **1** has been reported, and shows that compound **1** can be readily located in the compound **4** (Flavin adenine dinucleotide, FAD)-binding pocket of CRY2^53^. Compound **4** was also proven to be an endogenous ligand which can stabilise CRY proteins by competing with F-Box and leucine rich repeat protein 3 (FBXL3), thus lengthening the circadian period^54^. The complex of small molecule and protein is vital in understanding the binding mode and further improving the potency for acting as a modulator against protein. Therefore, the highly active compound 2-(9H-carbazol-9-yl)-N-(2-chloro-6-cyanophenyl)acetamide (compound **5**) was disclosed under structure–activity relationship analysis and CRY2-compound **1** complex structure^55^. Compound **5** can lengthen the circadian period, repress Per2 activity, and stabilise CRY better than compound **1**. More interestingly, another group discovered a series of compound **1** derivatives, compounds **6**–**8**, which can shorten the period by targeting cryptochrome in the mammalian circadian clock^56^. Unfortunately, no physiological effects were reported by subsequent studies. The novel derivative of 2-ethoxypropanoic acid, compound **9**, can inhibit the target CRY1 and CRY2^57^. Compound **9** can enhance E-box-mediated transcription and attenuate the rhythm without affecting the period. Recently, the potent compound 1–(3-(3,6-difluoro-9H-carbazol-9-yl)-2-hydroxypropyl)imidazolidin-2-one (compound **10**) significantly enhanced glucose clearance at 100 mg/kg in an oral glucose tolerance test^58^. Furthermore, the compound N-(2–(2,4-dimethylphenyl)-2,6-dihydro-4H-thieno[3,4-c]pyrazol-3-yl)-3,4-dimethylbenzamide (compound **11**) as a selective agonist for CRY1 and 1–(4-chlorophenyl)-N-(2–(4-methoxyphenyl)-5,5-dioxido-2,6-dihydro-4H-thieno[3,4-c]pyrazol-3-yl)cyclopentane-1-carboxamide (compound **12**) as moderately selective agonist for CRY2 than CRY1 were reported by using human U2OS cells with a Bmal1 promoter-luciferase (Bmal1-dLuc) reporter^59^. The X-ray crystal structures of CRY1 in complex with compound **11** and compound **12** show that these molecules were located in the FAD-binding pocket. As a useful tool for high selectivity against CRY isoform, the compound **11** and compound **12** were proved to facilitate brown adipocyte differentiation. Altogether, the modulators including the agonist or inhibitor of CRYs may be useful tools to treat circadian clock-related diseases through its action on CRY (see Figure 3 and Table 1).

**Table 1.** Modulators targeting CRYs.

Name	Activity	Actions	Physiological effects	Reference
**KL001** (Compound **1**)	IC_50_ = 14μM/0.82μM (measured by *Bmal1-dLuc* and *Per2-dLuc* reporter U2OS cells, Agonist)	Stabilise CRY, lengthen period, reduce amplitude	Inhibit glucagon-induced gluconeogenesis in primary hepatocytes	Hirota et al.^52^, Nangle et al.^53^
**KL002** (Compound **2**)	IC_50_ = 5.9μM/1.2μM (measured by *Bmal1-dLuc* and *Per2-dLuc* reporter U2OS cells, Agonist)	Stabilise CRY, lengthen period, reduce amplitude	Inhibit glucagon-induced gluconeogenesis in primary hepatocytes	Hirota et al.^52^
**KL003** (Compound **3**)	IC_50_ = 4.4μM/0.66μM (measured by *Bmal1-dLuc* and *Per2-dLuc* reporter U2OS cells, Agonist)	Stabilise CRY, lengthen period, reduce amplitude	Inhibit glucagon-induced gluconeogenesis in primary hepatocytes	Hirota et al.^52^
**FAD** (Compound **4**)	/	Stabilise CRY proteins by competing with FBXL3, lengthen the circadian period	Light-independent mechanisms of FAD regulate CRY	Hirano et al.^54^
**KL044** (Compound **5**)	log(EC_50_[M]) = –7.32 (Agonist)	Lengthen the circadian period, repress Per2 activity, and stabilise CRY	Inhibit glucagon-induced gluconeogenesis	Lee et al.^55^
**GO044** (Compound **6**)	/ (Inhibitor)	Shorten period	/	Oshima et al.^56^
**GO200** (Compound **7**)	/ (Inhibitor)			Oshima et al.^56^
**GO211** (Compound **8**)	/ (Inhibitor)			Oshima et al.^56^
**KS15** (Compound **9**)	EC_50_=0.49μM (Inhibitor)	Attenuate circadian oscillation, inhibit the repressive function of CRY1/2	Enhance E-box-mediated transcription	Chun et al.^57^
**50** (Compound **10**)	EC_50_ = 0.363μM (measured by *Per2-dLuc* reporter U2OS cells, Agonist)	Lengthen the circadian period, repress Per2 activity, and stabilise CRY	Inhibit glucagon-induced gluconeogenesis	Humphries et al.^58^
**KL101** (Compound **11**)	log[EC_2h_] = –5.79 (measured by Bmal1-dLuc cells, Agonist)	Stabilise CRY1 and lengthen period	Enhance brown adipocyte differentiation	Miller et al.^59^
**TH301** (Compound **12**)	log[EC_2h_] = –6.03 (measured by Bmal1-dLuc cells, Agonist)	Stabilise CRY1/2 and lengthen period	Enhance brown adipocyte differentiation	Miller et al.^59^

#### 2.1.2. Modulators for REV-ERBs

*Endogenous ligands for REV-ERBs.* In 2007, compound **13** was confirmed as a physiological ligand of nuclear receptors REV-ERBα (encoded by nuclear receptor subfamily 1, group D, member 1 (NR1D1)) and REV-ERBβ (Nuclear receptor subfamily 1, group D, member 2 (NR1D2)) by two research groups, Rastinejad et al. and Lazar et al^60^^,^^61^. Multiple biochemical and biophysical methods were used to demonstrate the association of compound **13** with ligand-binding domains of REV-ERB receptors, including mutation studies, transcriptional repressor function and repression of target gene transcription, ultraviolet-visible spectroscopy, mass spectrometry, isothermal titration calorimetry (ITC), and circular dichroism. Soon afterward, the crystal structure of REV-ERBβ in complex with compound **13** was also reported^62^^,^^63^. All the results disclosed suggest that compound **13** can bind the REV-ERBs and is indeed a physiological ligand of nuclear receptors REV-ERBs. In mammalian cells, compound **13** can cause the recruitment of the co-repressor nuclear receptor corepressor-1 (NCoR) by targeting REV-ERB, giving rise to the repression of target genes including BMAL1 (also known as ARNTL)^60^. Moreover, by targeting the REV-ERBα, compound **13** can suppress the expression of hepatic gluconeogenic gene and the output of glucose^61^. These findings would facilitate the development of small molecule modulators against REV-ERBs to treat diseases related to the dysfunctional disorder of metabolism and the mammalian clock.

**Figure 4.**
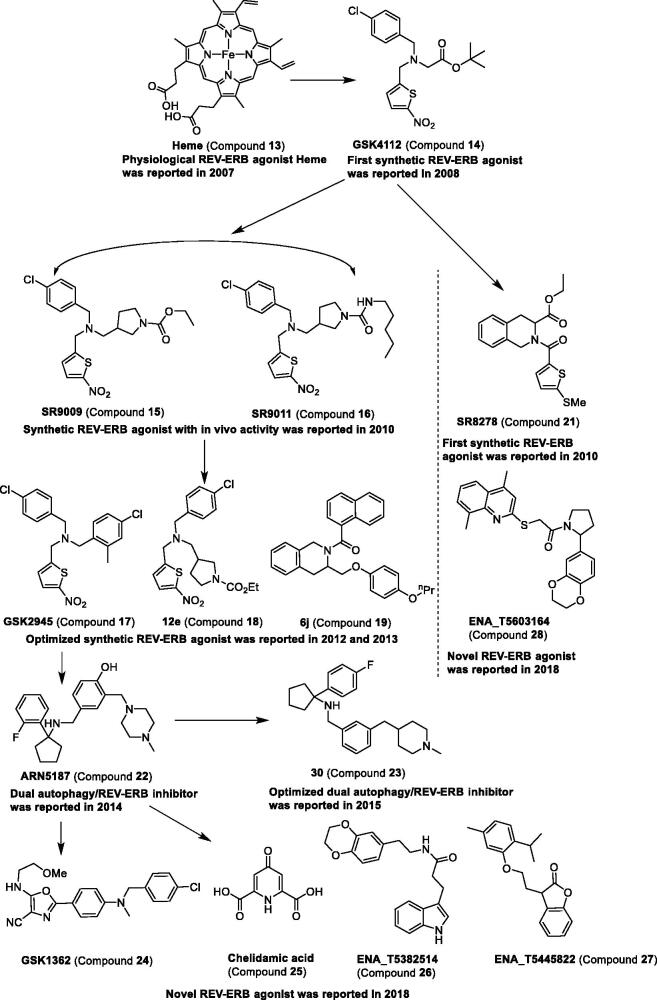
Development and structure of modulators targeting REV-ERBs.

*Synthetic ligands for REV-ERBs.* In 2008, the compound 1,1-dimethylethyl N-[(4-chlorophenyl)methyl]-N-[(5-nitro-2-thienyl)methyl]glycinate was reported by using REV-ERBα–NCoR fluorescence resonance energy transfer (FRET) assay, which showed an EC_50_ value of 250 nM^64^. This compound was the first agonist of REV-ERBα and was competitive with compound **13**. In subsequent studies, this compound was successively named **SR6452** or **GSK4112** (compound **14**) (Table 2 and Figure 4)^65^^,^^66^. Compound **14** can induce adipocyte differentiation in 3T3-L1 cells, enhance the recruitment of nuclear receptor co-repressor (NCoR) to REV-ERBα, and inhibit expression of the circadian target gene *Bmal1*. In addition, similar to compound **13**, compound **14** also repressed the expression of gluconeogenic genes in liver cells and reduced glucose output in primary hepatocytes^66^. These studies suggest that compound **14** may be used to treat diabetes or to modulate the circadian rhythm.

**Table 2.** Modulators targeting REV-ERBs.

Name	Activity	Actions	Physiological effects	Reference
**Heme** (Compound **13**)	IC_50_ = 0.05μM (measured by FRET assay, agonist)	Represses activity of REV-ERBα LBD	Regulates interaction between REV-ERBα and NCoR-HDAC3	Raghuram et al.^60^, Yin et al.^61^
**GSK4112**/**SR6452** (Compound **14**)	EC_50_ = 0.25μM (measured by FRET assay, agonist)	Resets the circadian oscillation of REV-ERB target genes, suppresses expression of REV-ERB target genes in cells	Inhibits expression of the circadian target gene *bmal1*	Meng et al.^64^, Kumar et al.^65^ Grant et al.^66^
**SR9009** (Compound **15**)	IC_50_ = 0.67/0.80μM (measured by Gal4 reporter assay for REV-ERBα and REV-ERBβ, agonist) K_d_ = 0.8μM (measured by circular dichroism binding assay for REV-ERBα)	Amplitude reduction, suppresses RRE-mediated transcription	Improves glucose homeostasis in obese mice, promotes wakefulness, reduces anxiety	Solt et al.^67^
**SR9011** (Compound **16**)	IC_50_ = 0.79/0.56μM (measured by Gal4 reporter assay for REV-ERBα and REV-ERBβ, agonist)	Amplitude reduction, suppresses RRE-mediated transcription	Improves glucose homeostasis in obese mice, promotes wakefulness, reduces anxiety	Solt et al.^67^
**GSK2945** (Compound **17**)	EC_50_ = 0.05μM (measured by NCOR peptide recruitment for REV-ERBα, agonist)	Suppression and shift of the BMAL oscillation curve	Inhibits IL-6 production from human THP-1 cells	Trump et al.^68^
**12e** (Compound **18**)	EC_50_ = 0.7μM (measured by full-length *Bmal1* reporter assay for REV-ERBα, agonist)	Suppresses expression of REV-ERB target genes in cells	Inhibits expression of the circadian target gene *bmal1*	Shin et al.^69^
**6j** (Compound **19**)	EC_50_ = 0.077μM (measured by full-length *Bmal1* reporter assay for REV-ERBα, agonist)	Suppresses expression of REV-ERB target genes in cells	Inhibits expression of the circadian target gene *bmal1*	Noel et al.^70^
**KK-S6** (Compound **20**)	IC_50_ = 3.95μM (measured by cell-based assay using the wtBmal1: Luc-transfected NIH3T3 cells, agonist)	Alters the amplitude of circadian oscillations of *Bmal1* and *Per2*	Represses RORE-dependent transcriptional activity of *mBmal1* promoter and reduces endogenous BMAL1 protein expression	Lee et al.^71^
**SR8278** (Compound **21**)	IC_50_ = 0.47μM (measured using full-length *Bmal1* reporter assay for REV-ERBα, Antagonist)	Increases expression of REV-ERB target genes in cells	Reduces glucagon secretion from mouse alpha cells	Kojetin et al.^72^
**ARN5187** (Compound **22**)	IC_50_ = 17.5μM (measured using luciferase-based reporter assay, dual autophagy/REV-ERB inhibitor)	Direct interaction with the LBD of REV-ERBβ	Enhances the expression of *BMAL1*, *PER1*, and *PEPCK*, and blocks autophagy by disrupting the lysosomal function and preventing autophagolysosome final maturation	De Mei et al.^73^
**30** (Compound **23**)	IC_50_ = 1.34μM (measured using luciferase-based reporter assay, dual autophagy/REV-ERB inhibitor)	Direct interaction with the LBD of REV-ERBβ	Enhances the expression of *BMAL1*, *PER1*, and *PEPCK*, and blocks autophagy by disrupting the lysosomal function and preventing autophagolysosome final maturation	Torrente et al.^74^
**GSK1362** (Compound **24**)	inverse agonist	Protects REV-ERBα protein from degradation	Increases transcription of *Bmal1*	Pariollaud et al.^75^
**Chelidamic acid** (Compound **25**)	EC_50_ = 0.36μM (measured using mammalian cell-based two-hybrid system, agonist)	Binds specifically to the LBD site of REV-ERBα receptor	/	Hering et al.^76^

Although compound **14** was used as a probe to investigate the pharmacological effects in *in vitro*, it has a poor pharmacokinetic profile with rapid clearance (C_int_ > 1.0 ml min^−1 ^mg^−1^ protein) in rat liver microsomes and lower oral bioavailability (F ≤ 1% in mice)^66^. Therefore, a series of analogues of compound **14** were synthesised by medicinal chemists to explore the applicable pharmacokinetics and pharmacodynamics used in *in vivo* studies.

The analogues of compound **14**, the potent compounds** 15**–**16**, were disclosed by Burris et al.^67^, which were the first REV-ERB agonists with *in vivo* activity. Compounds **15**–**16** can generate loss of locomotor activity during the subject dark phase and 1–3-h delay in the onset of nocturnal locomotor activity. The two compounds can alter the expression of the core clock genes, including *Per2*, *Bmal1*, *Clock*, *Cry2*, and *Npas2*. The ability of REV-ERB agonists in modulating the circadian behaviour of C57BL/6 mice may be used as a drug to treat sleep disorders and jet lag. Indeed, compound **15** was found to be able to induce wakefulness and reduce paradoxical sleep-rapid eye movement (REM) and slow-wave sleep^77–79^.

As previously reported, the double-knockout REV-ERBα and REV-ERBβ mice can also markedly alter metabolic effects^80^. The administration of the agonist of REV-ERBα and REV-ERBβ, compound **16**, gives rise to increase in energy expenditure and weight loss^67^. In obese mice, including diet-induced obese mice and genetic model of obesity (OB/OB mice), REV-ERB agonist treatment results in a decrease in fat mass and plasma lipids. Recently, a study investigated further the metabolic profile of the nuclear receptor REV-ERB agonist. The results of the experiment show that the enzymatic isoforms mainly involved in the compound **15** phase I biotransformation pathways are cytochrome P450 3A4 (CYP3A4), cytochrome P450 3A5 (CYP3A5), cytochrome P450 2C19 (CYP2C19), and cytochrome P450 2D6 (CYP2D6)^81^.

With the further study of REV-ERB agonist, compound **15** was associated with heart failure^82^, cancer^83^^,^^84^, atherosclerosis^85^, chikungunya and O’nyong’nyong virus^86^, and autoimmune disease^87^. However, Lazar et al.^88^ discovered that compound **15** can decrease cell viability, rewire cellular metabolism, and alter gene transcription in hepatocytes and embryonic stem cells lacking both REV-ERBα and REV-ERBβ, which means that the effects of compound **15** cannot be used solely as surrogate for REV-ERB activity. Therefore, more efforts are needed to explore its mechanism of action. Highly selective compounds also need to be developed urgently.

According to published papers in the same period as compound **15**, Kamenecka et al. also conducted structure-activity relationship analysis on compound **14**. Compounds **18–19** show slightly better plasma and brain exposure as compound **14**, but they displayed the best CNS exposure with brain penetration of 100% or 67%, respectively^69^^,^^70^. The analogue of compound **14**, compound **17**, was reported by Tomkinson et al.^68^ Compound **17** shows > 1000-fold selectivity over liver X receptor α (LXRα) and is a potent agonist with REV-ERBα activity (EC_50_ = 0.05 μM), which may be the best compound with high selectivity and may serve as a pharmacological toolbox to investigate the biology of REV-ERBα. Recently, the novel small molecular compound **20** was disclosed, which can reinforce REV-ERBα activity by acting in a RORE-dependent manner, though not by the same mechanism as known REV-ERB agonists. It may also provide a new way of exploring the REV-ERB modulator^71^.

The first antagonist of REV-ERBα is compound **21** (Table 2 and Figure 4). Compound **21** is derived from compound **14** based on the tertiary amine scaffold. In HepG2 cells, compound **21** could increase the expression of either *glucose 6-phosphatase (G6Pase)* or *phosphoenolpyruvate carboxykinase (PEPCK)* mRNA expression by blocking the action of the endogenous agonist^72^. Compound **21** also caused significant increases in the expression levels of growth/differentiation factor *Growth and differentiation factor 10 (GDF10)* and *Growth and differentiation factor 15 (GDF15)* in uterine endometrial stromal cells (UESCs). These results show that cellular oscillators may serve an important role of regulating the expression of downstream genes during the differentiation of UESCs^89^.

Although the pharmacokinetic properties of small molecular compound **21** is poor^72^, which has also been confirmed by our group^90^, compound **21** serves as a useful probe to explore the REV-ERB function by others. In vesicular stomatitis virus (VSV)-induced encephalitis model, administration of compound **21** increased *C-C motif chemokine ligand 2 (CCL2)* mRNA expression and decreased mice survival, which is associated with neuroprotective effects and lifetime^91^. The molecular connection between the circadian timing system and mood regulation was identified by Kim et al.^92^ The circadian nuclear receptor REV-ERBα is associated with bipolar disorder, as it influences midbrain dopamine production and mood-related behaviour in mice. Treatment with compound **21** induced mania-like behaviour in association with a central hyperdopaminergic state. The evidence suggests that targeting REV-ERBα may be beneficial to the treatment of circadian rhythm-related affective disorders. Compound **21** could slow the progression of muscular dystrophy by increasing lean mass and muscle function and decreasing muscle fibrosis and muscle protein degradation in C57BL/10ScSn-Dmd^mdx^/J (mdx) mice^93^. This research suggests that the antagonist compound **21** of REV-ERB may be a profound agent for the treatment of Duchenne muscular dystrophy (DMD). In conclusion, these results suggest that compound **21** is a unique chemical tool. However, it must be clearly recognised that poor pharmacokinetic properties of compound **21** also limit the further development of the compound. It is urgent to discover novel and potent compounds against REV-ERBs.

The novel dual autophagy/REV-ERB inhibitor compound **22** was revealed in 2014^73^. Compound **22** can relieve the clock transcriptional repression mediated by REV-ERB and enhance the expression of REV-ERB target genes, *Bmal1*, *Per1*, and *phosphoenolpyruvate carboxykinase (PEPCK)*, in BT-474 cells. It can also block autophagy by disrupting the lysosomal function and preventing autophagolysosome final maturation. Although the potency of compound **22** is under micromolar range, this compound provides an uncloaking the new measures to treat cancers. Therefore, Grimaldi et al.^74^ carried out structure–activity relationship (SAR) studies of compound **22** and finally obtained the potent compound **23** (1–(4-Fluorophenyl)-N-[[3-[(1-methyl-4-piperidyl)methyl]phenyl]methyl]cyclopentanamine) with 15-fold greater REV-ERBβ-inhibitory and cytotoxic activities compared to compound **22**.

Recently, a novel oxazole inverse agonist of REV-ERB, compound **24**, was discovered by Ray et al.^75^ based on fluorescence resonance energy transfer (FRET) assay. Compound **24** showed a high selectivity over 20 nuclear receptors, which can reverse the degradation of REV-ERBα protein mediated by inflammatory stimuli. Subsequently, Gul et al.^76^ established a mammalian cell-based two-hybrid assay system and found compound **25** as a novel agonist of REV-ERB. In addition, three other compounds against REV-ERB, compounds **26–28** (Figure 4), were found using this method. Compound **28** was confirmed as an antagonist, and compounds **26**–**27** were confirmed as agonists. Although the three compounds showed a poor selectivity against other targets, these compounds present a new kind of scaffold and can be used as a profound hit to reveal a drug-like compound.

#### 2.1.3. Modulators for RORs

**Figure 5.**
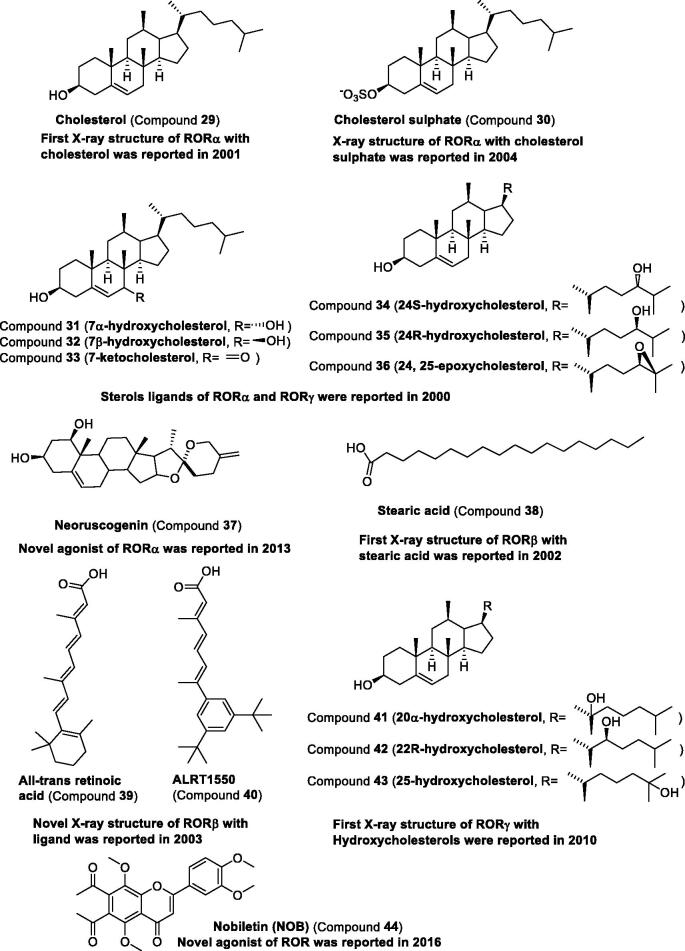
Natural structure of modulators targeting RORs.

*Natural ligands for RORs.* In 2002, the first ligand of RORα, compound **29**, was proved by X-ray structure (PDB entry 1N83). It is present in the ligand-binding pocket (LBP) and is important in designing the ligand targeting RORs^94^. The analogue of compound **29**, compound **30**, can also bind to RORα as confirmed by the crystal structure (PDB entry 1S0X)^95^. Other sterols including oxysterols as ROR inverse agonists and neoruscogenin as RORα agonist were found and reviewed in other papers^96^^,^^97^. The representative structure of sterols (compounds **31**–**37**) is presented in Figure 5 to analyse the structure for researchers. In 2001, the first ligand of RORβ, compound **38**, was proved by X-ray structure (PDB entry 1K4W)^98^. This crystal structure of compound **38** and the ligand-binding domain (LBD) of the rat RORβ shed new light on the development of ligands against RORs. Subsequently, the crystal structure of the complex between compound **38** and RORβ (PDB entry 1N4H) was solved by Schüle group^99^. They also solved the crystal structure of the complex between synthetic analog compound **40** and RORγ (PDB entry 1NQ7). All these two-crystal structures present similar results, namely, the compound **39** and analogs were binding to the RORβ ligand-binding domain (LBD). Hydroxycholesterols (compounds **41**–**43**) were binding to the RORγ LBD using the same method in 2010, with accession codes 3KYT (RORγ/Compound **41**), 3L0J (RORγ/Compound **42**), and 3L0L (RORγ/Compound **43**), respectively^100^. Recently, the natural compound **44** as an agonist for the ROR was reported by using *Clock*^Δ19/+^ cells with *PER2::Luc* reporter^101^^,^^102^. The potent natural compound and all these crystal structures of the complex between natural ligand and ROR have inspired researchers to search for potent and selective small molecule modulators targeting RORs (Figure 5).

**Figure 6.**
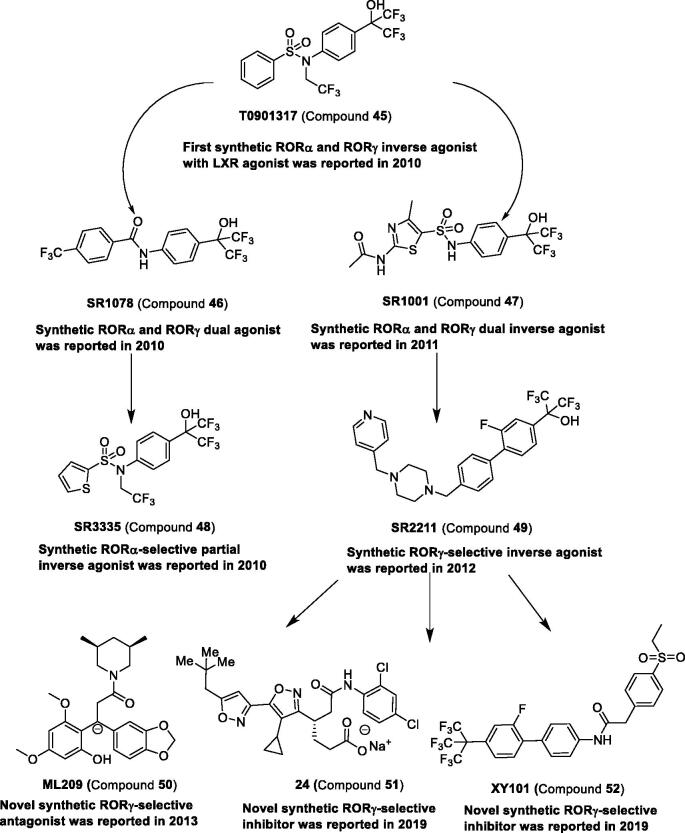
Development and structure of synthetic modulators targeting RORs.

*Synthetic ligands for RORs.* In 2010, using cell-based GAL4-NR LBD cotransfection assay, Griffin et al.^103^ found the first RORα/γ inverse agonist compound **45**, which was also the agonist of the liver X receptor (LXR)^104^. Compound **45** was binding to RORα/γ but not to RORβ. This compound provided the scaffold to further exploit the potent and selective ligands targeting ROR. A compound with multiple targets is not an ideal tool to disclose the function of protein. Therefore, the core scaffold of compound **45** was optimised, and a round of agonists or inverse agonists against RORα/γ, RORα, and RORγ were reported. These compounds have been reviewed elsewhere^96^^,^^97^. The representative compounds can be found in Figure 6 and Table 3 to systematically review the research studies.

**Table 3:** Representative modulators targeting RORs.

Name	Activity	Actions	Physiological effects	Reference
**T0901317** (Compound **45**)	K_d_ = 132 nM and 51 nM for RORα and RORγ (measured by radioligand displacement, inverse agonist)	Inhibits transactivation activity of RORα and RORγ but not RORβ	Suppresses *G6PC* and *IL1*7 promoter activity	Kumar et al.^103^
**SR1078** (Compound **46**)	IC_50_ = 2–5 μM for RORα and RORγ (measured by DualGloTM luciferase assay, agonist)	Decreases interaction between RORγ and the peptide fragment of TRAP220 co-activator	Increases the expression of RORα and RORγ target genes *in vitro* and *in vivo*	Wang et al.^105^
**SR1001** (Compound **47**)	K_i_ = 172 and 111 nM for RORα and RORγ (measured by radioligand binding assay, inverse agonist)	Inhibits RORγ activity on the *IL17* promoter	Inhibits expression of *IL17A, IL17F, IL21,* and *IL22* in cells	Solt et al.^106^
**SR3335** (**ML176**, Compound **48**)	K_i_ = 220 nM (measured by radioligand binding assay, partial inverse agonist)	Inhibits the constitutive transactivation activity of RORα	Suppresses *G6PC* and *PCK* promoter activity	Kumar et al.^107^
**SR2211** (Compound **49**)	K_i_ = 105 nM (measured by radioligand binding assay, antagonist)	Affects the structural conformation of RORγ LBD	Suppresses *IL17* expression, IL-17 production and TH17 cell differentiation	Kumar et al.^108^
**ML209** (Compound **50**)	IC_50_ = 0.5 μM for RORγ (measured by VP16 assay, inverse agonist)	Improves stabilisation effects for the RORγ protein	Suppresses human T_H_17 cell differentiation	Huh et al.^109^
**24** (Compound **51**)	EC_50_ = 9 nM for RORγ (measured by human RORγ luciferase (LUC) assay, inhibitor)	Improves transactivation activity of RORγ	Suppresses production of IL-17 *in vivo*	Kotoku et al.^110^
**XY101** (Compound **52**)	IC_50_ = 30 nM for RORγ (measured by cell-based reporter gene assay, inverse agonist)	Improves transactivation activity of RORγ and stabilisation effects for the RORγ protein	Suppresses cell growth, colony formation, and expression of AR, AR-V7, and PSA	Zhang et al.^111^

The first synthetic RORα-selective partial inverse agonist compound **48** based on the core scaffold of compound **45**^103^ and compound **46**^105^ was reported in 2010^107^. Compound **48** can inhibit the constitutive transactivation activity of RORα with an IC_50_ of 480 nM, but it cannot inhibit the activity of LXRα, RORβ, and RORγ. Compound **48** can suppress hepatic gluconeogenesis and improve glucose homeostasis *in vivo*, suggesting that compound **48** may be a potential tool to treat type 2 diabetes.

After structure–activity relationship (SAR) studies of compound **47**, the potent and selective inverse agonist compound **49** targeting RORγ was obtained, which can reduce the conformational mobility of RORγ LBD. The other potent and selective agonists, inverse agonists, or inhibitors of RORγ were reviewed elsewhere^50^^,^^112^. Recently, 4-(isoxazol-3-yl) butanoic acid derivatives as high selective inhibitors of RORγ were reported. The potent compound **51** showed commendable anti-inflammatory effects in a mouse dermatitis model. A novel compound **52**, 2–(4-(ethylsulfonyl)phenyl)-N-(2′-fluoro-4′-(1,1,1,3,3,3-hexafluoro-2-(trifluoromethyl)propan-2-yl)-[1,1′-biphenyl]-4-yl)acetamide, in complex with the RORγ ligand binding domain (LBD), was reported^111^. Compound **52** possess good metabolic stability and pharmacokinetic profile, and shows a significant tumour growth inhibition *in vivo*.

### 2.2. Small molecule modulators with other or unknown targets

Compounds targeting other proteins including kinase, epigenetic proteins, and others can also alter circadian characteristics. All these compounds are summarised as follows.

#### 2.2.1. Modulators for kinases

*Casein kinase 1 (CK1).* The casein kinase family comprises seven distinct genes encoding CK1 isoforms (α, α2, γ1, γ2, γ3, δ, and ε) in mammals^113^. CK1δ and CK1ε have been discovered to regulate the circadian clock, and their substrates are proved to be PER1, PER2, BMAL1, and CRYs^114^. CK1ε-selective inhibitor compound **53** can increase in period length, leading to about 1.2-h in synchronised Rat-1 (mPer1::luc) cells^115^. Afterward, compounds **54–60** (Table 4 and Figure 7) were also proven to lengthen the period in cultured cells and were reviewed in other papers ^49^^,^^116^. Recently, compound **61** was identified as a regulator to increase period length in mammalian cells and larval zebrafish assay^117^. Compound **62** lengthens the period through CK1 inhibition^118^. All these studies reveal that the role of CK1 is important in the regulation of circadian rhythm^119^.

Figure 7.Development and structure of synthetic modulators targeting kinases.

**Figure:**
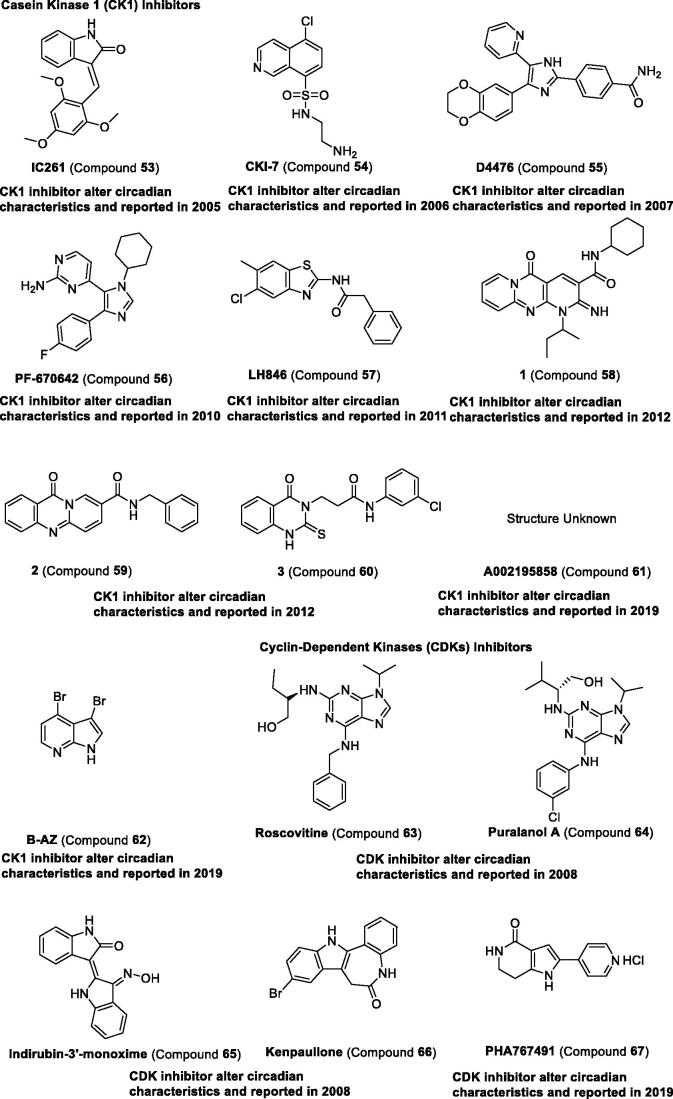

**Figure:**
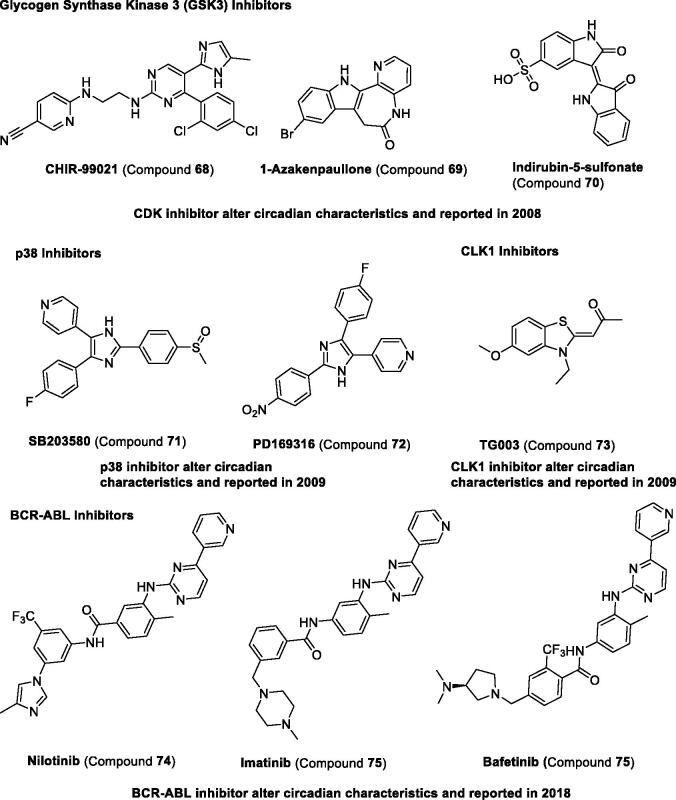

**Table 4:** Representative modulators targeting kinases.

Name	Activity	Physiological Effects	Reference
**IC261** (Compound **53**)	Inhibiting CK1ε	Period lengthening	Eide et al.^115^
**CKI-7** (Compound **54**)	Inhibiting CK1ε	Period lengthening	Vanselow et al.^116^
**D4476** (Compound **55**)	Inhibiting CK1ε	Period lengthening	Reischl et al.^120^
**PF-4800567** (Compound **56**)	Inhibiting CK1ε	Period lengthening	Meng et al.^121^
**LH846** (Compound **57**)	Inhibiting CK1δ	Period lengthening	Lee et al.^122^
**1-3** (Compound **58-60**)	Inhibiting CK1ε	Period lengthening	Chen et al.^102^
**A002195858** (Compound **61**)	Inhibiting CK1	Period lengthening	Mosser et al.^117^
**B-AZ** (Compound **62**)	Inhibiting CK1	Period lengthening	Ono et al.^118^
**Roscovitine** (Compound **63**)	Inhibiting CDK1, CDK2 and CDK5	Period lengthening	Hirota et al.^123^
**Puralanol A** (Compound **64**)	Inhibiting CDK2, CDK4 and CDK5	Period lengthening	Hirota et al.^123^
**Indirubin-3′-oxime** (Compound **65**)	Inhibiting CDK and GSK3	Period shortening	Hirota et al.^123^
**Kenpaullone** (Compound **66**)	Inhibiting CDK and GSK3	Period shortening	Hirota et al.^123^
**PHA767491** (Compound **67**)	Inhibiting CDK7/CDK9	Period lengthening	Uehara et al.^124^
**Chir99021** (Compound **68**)	Inhibiting GSK3β	Period shortening	Hirota et al.^123^
**1-azakenpaullone** (Compound **69**)	Inhibiting GSK3β	Period shortening	Hirota et al.^123^
**indirubin** (Compound **70**)	Inhibiting GSK3β	Period shortening	Hirota et al.^123^
**SB203580** (Compound **71**)	Inhibiting p38	Period lengthening	Isojimaa et al.^125^
**PD169316** (Compound **72**)	Inhibiting p38	Period lengthening	Isojimaa et al.^125^
**TG003** (Compound **73**)	Inhibiting CLK1	Period lengthening	Isojimaa et al.^125^
**Nilotinib** (Compound **74**)	Inhibiting BCR-ABL	Period lengthening	Tamai et al.^51^
**Imatinib** (Compound **75**)	Inhibiting BCR-ABL	Period lengthening	Tamai et al.^51^
**Bafetinib** (Compound **76**)	Inhibiting BCR-ABL	Period lengthening	Tamai et al.^51^

*Cyclin-dependent kinases (CDKs).* The cyclin-dependent kinase family comprises 11 distinct genes encoding CDK isoforms (1–11)^113^. CDK5 has been reported to directly phosphorylate CLOCK^126^, inhibitor compound **63** targets CDK1, CDK2, and CDK5, and compound **64** targets CDK2, CDK4, and CDK5 can lengthen the circadian period^123^. However, the multi-target inhibitors, compounds **65–66** targeting both CDK and GSK3, were proved to shorten the circadian period. Recently, compound **67**, an inhibitor of CDK7 and CDK9, has been reported to increase period length in mammalian cells^124^.

*Other kinases*. As other studies in the literature have reported, glycogen synthase kinase 3β (GSK3β) can also regulate the circadian clock, which can phosphorylate CLOCK, PER, REV-ERB, and CRY proteins^114^. The selective GSK3β inhibitors, compounds **68**–**70**, have been reported to shorten the circadian period^123^. Other kinase inhibitors including compounds **71–72** targeting p38 and compound **73** targeting CLK1 have been reported to increase period length^125^. Recently, compounds **74**–**76** selective BCR-ABL tyrosine kinase inhibitor were found to shorten the circadian period^51^ (Table 4 and Figure 7).

#### 2.2.2. Modulators for epigenetic proteins and others

Silent information regulator 1 (SIRT1) has been found to contribute to circadian control, which regulates circadian clock gene expression through PER2 deacetylation^36^^,^^127^. SIRT1 activator compound **77** is involved in physiological rhythms and clock gene expression^128^. The potent SIRT1 activators, compounds **78–81**, also show that they can reduce circadian expression, lengthen period, and reduce amplitude^128^. Recently, SIRT6 was also found to regulate circadian rhythms via Per2^129^. However, the small molecules of SIRT6 have not been tested by researchers.

**Figure 8.**
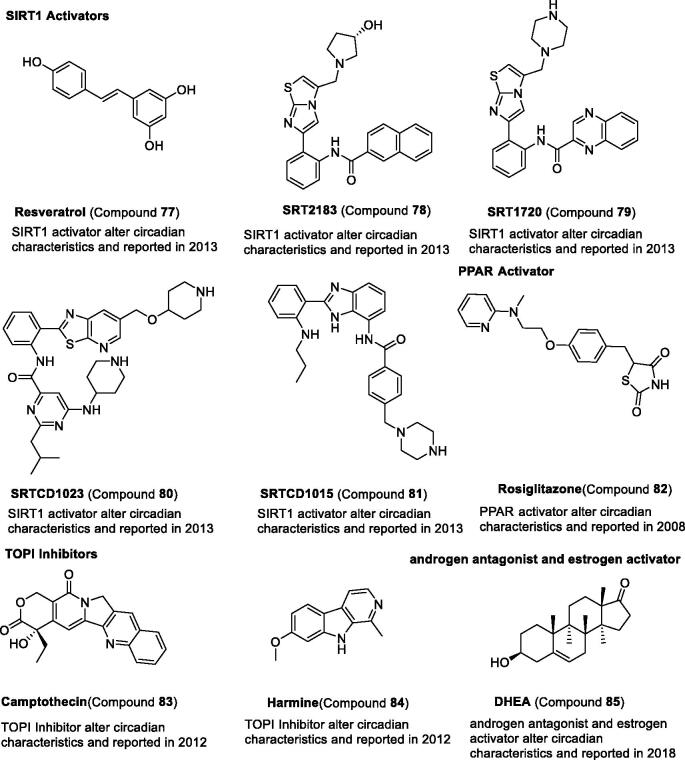
Development and structure of synthetic modulators targeting epigenetic proteins and others.

In a recent study, peroxisome proliferator-activated receptor γ (PPARγ) was involved in regulating the expression of *Bmal1* and *REV-ERB*α, and its agonist compound **82** can induce expression of *Bmal1*^131^. Compounds **83–84**, DNA topoisomerase (TOP) inhibitors, were also found to enhance the circadian expression and lengthen the circadian period^132^. Recently, the androgen antagonist and oestrogen activator compound **85** was found to shorten the circadian period^51^ (Table 5 and Figure 8).

**Table 5.** Representative modulators targeting epigenetic proteins.

Name	Activity	Physiological effects	Reference
**Resveratrol** (Compound **77**)	SIRT1 activator	Modulate physiological rhythms and clock gene expression	Chang et al.^128^
**SRT2183** (Compound **78**)	SIRT1 activator	Reduce circadian expression Lengthen period Reduce amplitude	Bellet et al.^130^
**SRT1720** (Compound **79**)	SIRT1 activator	Reduce circadian expression Lengthen period Reduce amplitude	Bellet et al.^130^
**SRTCD1023** (Compound **80**)	SIRT1 activator	Reduce circadian expression Lengthen period Reduce amplitude	Bellet et al.^130^
**SRTCL1015** (Compound **81**)	SIRT1 activator	Reduce circadian expression Lengthen period Reduce amplitude	Bellet et al.^130^
**Rosiglitazone** (Compound **82**)	PPARγ agonist	induce expression of *Bmal1*	Wang et al.^131^
**Camptothecin** (Compound **83**)	TOPI inhibitor	Enhance the circadian expression and lengthen the circadian period	Onishi et al.^132^
**Harmine** (Compound **84**)	TOPI inhibitor	Enhance the circadian expression	Onishi et al.^132^
**DHEA** (Compound **85**)	Androgen antagonist and oestrogen activator	Shorten the circadian period	Tamai et al.^51^

## 3. Implications in circadian rhythm-related diseases

**Figure 9.**
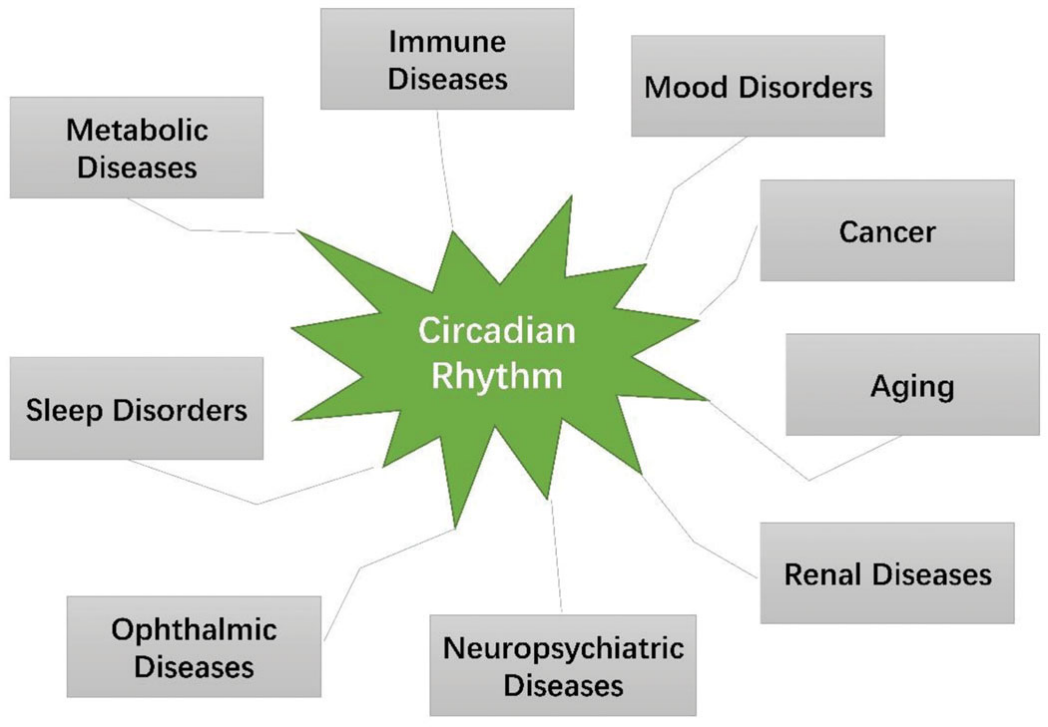
Implications in circadian rhythm-related diseases.

Circadian rhythm plays a very important role in the normal maintenance of organisms, but physical and psychological influences including jet lag, shift work, and diseases can cause a misalignment of the intrinsic oscillators. Jet lag occurs in individuals travelling across multiple time zones, who may suffer from some symptoms including disruption of sleep, gastrointestinal disturbances, decreased vigilance and attention span, a general feeling of malaise, and an increased risk of cancer and heart disease^133^^,^^134^. Shift work is apparent among people employed in factories or social event firms and work from 7 pm to 9 am^135^. Shift work has become a common phenomenon in society, and was found to be involved in cancer, cardiovascular disease, depression, and infertility. Jet lag and shift work induce rhythm disorder, which can cause a mass of psychological, nervous system, mental health, and physical health problems^135^. Beyond all that, diseases are closely related to circadian rhythms. Diseases can cause disturbances in circadian rhythms, and disorder in circadian rhythms, in turn, further aggravate the severity of the disease^136^^,^^137^. This section will focus on the relationship between disease and circadian rhythm disorders (Figure 9).

### 3.1. Metabolic diseases

Circadian rhythm has been associated with homeostasis and physiology, which is closely related to physical health^138^. Numerous lines of evidence are emerging that circadian dysfunctions are closely associated with increased risk for metabolic disease such as obesity and diabetes^136^^,^^139^. Evidence that the circadian rhythm is associated with energy homeostasis, glucose homeostasis, and lipid homeostasis has been found. Homozygous *Clock* mutant mice can lead to type 2 diabetes mellitus, with metabolic syndromes of hyperleptinemia, hyperlipidaemia, hepatic steatosis, and hyperglycaemia, with insufficient compensatory insulin production. *Clock* mutant animals can induce obesity, hyperphagia, reduced energy expenditure, adiposity, as well as dysregulation of glucose and lipid metabolism^140^. The core clock genes *Clock^mut^* or *Bmal1^−/−^* depress and abolish gluconeogenesis^141^. *Clock^mut^* also induced hypertriglyceridaemia in animal models^140^. REV-ERBα knockout mice also displayed altered lipid and bile metabolism^142^. Subsequent studies have shown that double knockout mice (REV-ERBα and REV-ERBβ) have disorganised lipid homeostatic gene networks^80^. The other core circadian rhythm gene *ROR* also turns out to be related to the regulation of energy homeostasis and several lipid and glucose metabolic genes^143^. Mutant RORα mice (also known as staggerer mice) display hypo-α-lipoproteinemia^144^. Recent studies have shown that RORα accommodates peripheral glucose tolerance, torpor, and hepatic lipid metabolism by regulating the expression of fibroblast growth factor 21 (FGF21)^145^^,^^146^. All of these pieces of evidence suggest that the circadian rhythm is associated with metabolism and that clock proteins can be as drug targets to treat metabolic diseases.

Many small molecule modulators of circadian proteins have been found to be useful in metabolic diseases. The CRYs activator compound **1** has been shown to inhibit glucagon-induced gluconeogenesis, which may provide a foundation for the treatment of diabetes^53^^,^^147^. Aside from the ligands of CRYs, the ligands of circadian nuclear receptors REV-ERB and RORs also demonstrated that they can be conducive to regulate metabolism *in vivo*. Compounds **15**–**16** as agonists of REV-ERBα and REV-ERBβ proved highly effective in the improvement of the metabolic profile in obese mice^67^. Recently, Chen et al.^101^ identified that compound **44** as an agonist for ROR can potently protect against metabolic syndrome and remodel the circadian and metabolic gene expression in diet-induced obese mice. Subsequently, they demonstrate that compound **44** can serve as a potential drug to treat the metabolic disorders and age-related decline by regulating cholesterol and bile acid metabolism^148^ and overcome the metabolic challenge by enhancing mitochondrial respiration in skeletal muscle^149^. Therefore, with an in-depth study of the mechanism for clock proteins and the discovery of selective and potent small molecule modulators, it is believed that in the near future, the ligands of CRYs, REV-ERBs, or RORs will provide first-class treatment for metabolic diseases such as obesity and diabetes.

### 3.2. Sleep disorders

Sleep plays a very important role in the biological process of all creatures; it is regulated by circadian rhythm and homeostatic mechanisms^150^. Normal circadian rhythms play an irreplaceable role in sleep. Circadian misalignments such as jet lag, shift work, and sleep deprivation have resulted in sleep disorders^134^^,^^135^. Kiessling’s group and Yamaguchi’s group identified that the different organs of mice showed heterogeneity entrainment kinetics in an experimental paradigm for jet lag^151^^,^^152^. The rhythm gene has been linked to sleep disorders. Mutations in both *PER2* (PER2 S662G) and *CSNK1D* (CK1δ T44A) have been involved in familial advanced sleep phase syndrome (FASPS)^153^. Recent studies indicate that the core clock gene expression has a close association with sleep apnoea (SA). Canales et al.^154^ identified that the Per3 expression of SA was lower than that in the normal group. Pharmacological treatment targeting the mammalian clock has been shown to have beneficial effects on sleep architecture^78^. Compound **16** as an agonist of REV-ERBα and REV-ERBβ displays increase in wakefulness and reduction of paradoxical sleep-rapid eye movement (REM) sleep and slow-wave sleep *in vivo*^67^^,^^78^. Therefore, the REV-ERB ligands may be beneficial in treating sleep disorders.

### 3.3. Ophthalmic diseases

As widely appreciated, light has profoundly influenced the mammalian circadian rhythm. Light is mainly received by intrinsically photosensitive retinal ganglion cells (ipRGCs)^155^. A large number of studies show that the knockout of the rhythm gene affects retinal processing of light information^156–160^. The circadian rhythm is involved in ophthalmic diseases including glaucoma, macular degeneration, cataract, retinitis pigmentosa, diabetic retinopathy, and optic nerve atrophy. Evidence is accumulating that glaucoma directly damages the light input into the circadian system and causes optic nerve dysfunction^161^^,^^162^. Recently, a mass of transcripts of nocturnal rodents and diurnal primates with daily and circadian oscillations were presented by RNA Sequencing (RNA-Seq) technology^163–165^. Panda et al.^163^ identified that around 4–12% of the transcripts are rhythmic in the cornea, optic nerve head, retina, and retinal pigment epithelium for young male baboons (*Papio anubis*). Recently, we also disclosed that 3% and 24% of the transcripts are rhythmic in the murine extraorbital lacrimal glands and murine cornea^164^^,^^165^. In addition, FitzGerald et al. identified that structural modification of the cornea and the lens was observed in *Bmal1* knockout mice^157^^,^^158^. Moreover, rhythm disorders can further aggravate diabetic retinopathy in *per2* knockout mice^160^. In previous studies, we found that compound **21** as an antagonist of REV-ERBα can enhance corneal wound healing^166^. Therefore, the small molecule modulators of circadian proteins provide a potential solution for the treatment of ophthalmic diseases.

### 3.4. Other diseases

The impact of the circadian system on immune diseases^166^^,^^167^, mood disorders^168^^,^^169^, neuropsychiatric diseases^18^^,^^170^, aging^171^^,^^172^, renal diseases (such as hypertension, chronic kidney disease, renal fibrosis, and kidney stones)^173^^,^^174^, and cancer^175^^,^^176^ has been reviewed by others. As described in section 2, small molecule modulators of circadian proteins supply pharmacological tools to treat these diseases. For example, the REV-ERB ligand compound **14** can regulate innate immune responses by repressing *interleukin 6 (il6)*^92^. Interestingly, Kim et al. identified that the pharmacological inhibition of REV-ERBα activity produces mania-like behaviour. The mice showed more hyperactive behaviour after the administration of REV-ERBα antagonist compound **21**^177^. The REV-ERBα agonist may be useful for mood regulation.

## 4. Perspectives and concluding remarks

In this review, we detailed all aspects of the physiological basis, molecular clock loops, biological function, potential targets, and small molecule modulators of circadian rhythm. The generation, maintenance, and regulation of circadian rhythms depend on the synergy of the circadian clock system, circadian input system, and circadian output system at the overall level. In particular, the circadian clock system is composed of the central circadian clock and the peripheral circadian clock. The apex of this system is the SCN master pacemaker in mammals. The periodic oscillation of circadian rhythm depends on the precise regulation of the circadian clock gene and the clock-controlled gene regulatory network, including transcriptional-translational feedback loops and the non-transcription mechanism of post-translational modification.

Extensive research has been performed on the relationship between circadian clock disorder and disease. Circadian clock genes knockout has confirmed that circadian misalignment is involved in metabolic syndrome, cardiovascular diseases, acute lung injury and inflammation, neurological diseases, immune diseases, cancer, mood disorders, sleep disorders, and ophthalmic diseases. As summarised in this article, circadian rhythms are important for human health, which suggests that the development of small molecules is imminent and could be used to treat circadian rhythm related diseases.

More importantly, a large number of small molecule modulators of circadian rhythm have been discovered, and most modulators have potential therapeutic effects on disease. In order to identify hits of the circadian clock, hundreds of thousands of compounds have been filtered by cell-based high-throughput circadian assays. The effectiveness of chemical biology approaches contributed to the discovery of the small molecule modulators of circadian rhythm^178^. In recent years, with the emergence and popularisation of some new technologies, biophysical methods (such as differential scanning fluorimetry, differential scanning calorimetry, isothermal titration calorimetry, and surface plasmon resonance) and computer-aided drug design will help in the discovery of more modulators targeting clock proteins. It is believed that in the near future, small molecule modulators will be a useful tool in the treatment of circadian rhythm related diseases.
